# Fetal Deaths and Proximity to Hazardous Waste Sites in Washington State

**DOI:** 10.1289/ehp.9750

**Published:** 2007-02-14

**Authors:** Beth A. Mueller, Carrie M. Kuehn, Carrie K. Shapiro-Mendoza, Kay M. Tomashek

**Affiliations:** 1 Public Health Sciences, Fred Hutchinson Cancer Research Center, Seattle, Washington, USA; 2 Department of Epidemiology, School of Public Health & Community Medicine, University of Washington, Seattle, Washington, USA; 3 Centers for Disease Control and Prevention, Division of Reproductive Health, Maternal and Infant Health Branch, Atlanta, Georgia, USA

**Keywords:** birth certificates, environmental exposures, fetal death, fetal death certificates, pesticides

## Abstract

**Background:**

The *in utero* period is one of increased susceptibility to environmental effects. The effects of prenatal exposure to environmental toxicants on various adverse pregnancy outcomes, including fetal death, are not well understood.

**Objective:**

We examined the risk of fetal death in relation to maternal residential proximity to hazardous waste sites.

**Methods:**

We conducted a population-based case–control study using Washington State vital records for 1987–2001. Cases were women with fetal deaths at ≥ 20 weeks (*n* = 7,054). Ten controls per case were randomly selected from live births. Locations of 939 hazardous waste sites were identified from the Department of Ecology registry. We measured distance from maternal residence at delivery to the nearest hazardous waste site, and calculated odds ratios (ORs) and 95% confidence intervals (CIs).

**Results:**

The risk of fetal death for women residing ≤ 0.5 miles, relative to > 5 miles, from a hazardous waste site was not increased (adjusted OR = 1.06; 95% CI, 0.90–1.25). No associations were observed for any proximity categories ≤ 5 miles from sites with contaminated air, soil, water, solvents, or metals; however, fetal death risk increased among women residing ≤ 1 mile from pesticide-containing sites (OR = 1.28; 95% CI, 1.13–1.46).

**Conclusion:**

These results do not suggest that fetal death is associated with residential proximity to hazardous waste sites overall; however, close proximity to pesticide-containing sites may increase the risk of fetal death.

More than 6 billion tons of waste are produced annually in the United States and are stored at more than 15,000 hazardous waste sites (National Resource Council on Environmental Epidemiology 1991). Most of these are waste-storage or treatment sites such as landfills or sites formerly used by industries (Landrigan et al. 1999). To address the health hazards associated with exposure to the contents of these sites, in 1980, the U.S. Congress enacted the Comprehensive Environmental Response, Compensation, and Liability Act, more commonly referred to as the Superfund Act [National Resource Council on Environmental Epidemiology 1991; U.S. Environmental Protection Agency (EPA) 2006b]. The act was amended in 1986 to provide additional funds, emphasize permanent remediation, increase state involvement, and improve efforts to deal with human health problems associated with proximity to these sites. The National Research Council Committee on Environmental Epidemiology (1991) reported that in 1991, 40 million people lived within 4 miles of a Superfund site.

One of the concerns about living near hazardous waste sites is the effect it may have on fetal development. The *in utero* period is one of increased susceptibility to environmental effects, and some studies have suggested that prenatal exposure to environmental toxicants may result in spontaneous abortion, malformations, or low birth weight (Carpenter 1994; Landrigan et al. 1999). Results of some other studies, however, have not been consistent with these observations (Baker et al. 1988; Croen et al. 1997; Fielder et al. 2000; Kharrazi et al. 1997; Sosniak et al. 1994).

Reducing the mortality ratio (the number of fetal deaths per 1,000 live births) among fetuses of ≥ 20 weeks gestation to 4.1 deaths per 1,000 live births has been identified as a national public health priority by the U.S. Department of Health and Human Services (2000). In 2003, the fetal mortality ratio in Washington State was 6.2 (Washington State Department of Health, Center for Health Statistics 2006). We used population-based data on fetal deaths of ≥ 20 weeks gestation and live births, in combination with geographical information systems (GIS) techniques, to examine the relationship of fetal death and maternal residential proximity during pregnancy to hazardous waste sites overall, by type of contaminated media and by hazardous substance present in the waste sites.

## Methods

Institutional review board approval was granted by the Washington State Department of Health and the Fred Hutchinson Cancer Research Center before the study was begun. Using birth and fetal death records from the Washington State Department of Health, potential cases for our study were identified from all fetal death records during 1987–2001 (*n* = 7,095). Ten potential controls were randomly selected from among records of live-born infants during the same years (*n* = 70,950). After we excluded those with gestational age < 20 weeks (the state’s definition of reportable fetal death), there were 7,054 cases and 70,938 controls remaining for analysis. Vital records data included parental characteristics, variables related to the pregnancy and the mother’s reproductive history and chronic medical conditions, delivery complications, source of payment for medical care, infant/fetal conditions such as weight and presence of malformations, the mother’s residence at the time of the live birth or fetal death, and length of time she had lived at that residence. The geocoordinates (latitude and longitude) of maternal residences were derived by the Washington State Department of Health, Division of Information Resource Management using Centrus and ArcView software (Washington State Department of Health Division of Information Resource Management 2006). In this process, many different street centerline and parcel databases were used to enhance the accuracy and completeness of address matching. Geocodes were obtained for 5,302 cases and 61,455 controls (75% and 86% of the eligible subjects in each group).

Washington currently has 46 National Priority List (NPL), or “Superfund,” sites and 17 sites that have already been cleaned up and deleted from the NPL (U.S. EPA 2006a). In 1988, Washington passed the Model Toxics Control Act (MTCA), which gave the Washington State Department of Ecology authority to clean hazardous waste sites and improve hazardous waste site management (Minnick et al. 2000). The potential hazard to public or environmental health of each site is rated on a scale of 1–5 in accordance with the Washington Ranking Model (WARM); NPL sites receive a ranking of 0 under the WARM method.

We obtained hazardous waste site data from the Washington State Department of Ecology Confirmed and Suspected Contaminated Sites (CSCS) Report (Washington State Department of Ecology Toxics Cleanup Program 2002). MTCA sites with WARM rankings and NPL Superfund sites listed on the 2000 CSCS were used for analysis in this study (*n* = 939). These data were publicly available at the time of our analysis, and were accessed in spreadsheet format from the Department of Ecology website (Washington State Department of Ecology Toxics Cleanup Program 2002). Geocoordinates were provided for each hazardous waste site in the CSCS data set. Coordinates within this database were calculated using a variety of methods including aerial photography, global positioning systems, address matching, 1990 census block and ZIP code centroids.

Using Maptitude software (version 4.5; Caliper Corporation, Newton, MA), we measured the straight-line distances in miles between the mother’s residence at the time of the live delivery or fetal death and the nearest hazardous waste site, the nearest “high-priority” waste site (defined as any Superfund site or any MTCA site with a WARM ranking of 1 or 2), and the nearest “low-priority” waste site (WARM ranking of 3–5). We treated these distances as categorical variables (within 0.5 miles, > 0.5 to 1 mile, > 1 mile to 2 miles, > 2 miles to 5 miles, > 5 miles) and collapsed categories as necessary in some subanalyses for which data were limited.

To estimate the relative risk of fetal death associated with residing at various distances from hazardous waste sites, we first conducted stratified analyses using Mantel-Haenszel risk estimators. We then used odds ratio (OR) estimates of the relative risk from multivariable logistic regression. Variables examined for their possible effects on the relationships of interest included maternal and paternal age, maternal race/ethnicity, parity, gravidity, prenatal smoking and alcohol consumption, urban/rural residence, pregnancy complications such as hypertension and diabetes, and indicators of socioeconomic status such as maternal educational level and use of Medicaid to pay for pre-natal care. We adjusted only for those variables that meaningfully altered the OR (generally by at least 10%). Unless otherwise indicated, all analyses are adjusted for maternal age, prenatal smoking status, and number of prior pregnancies. We also conducted subanalyses using cut points previously used in studies examining the timing of fetal death [< 28 weeks (early) and ≥ 28 weeks gestation (late)] (Bech et al. 2005; Tomashek et al. 2006), by the types of hazardous substances (solvents, metals, pesticides, radioactive substances) and contaminated media (water, drinking water, soil and sediment, air) at the nearest site, and the duration of time that mothers lived at their residence before the delivery or fetal death. Evaluations of specific site types (e.g., high priority) included assessment of the possible effects of proximities to the other types as well, to try to isolate any effects. Analyses were conducted with STATA statistical software, version 8 (StataCorp., College Station, TX).

## Results

The distributions of women whose pregnancies ended in fetal deaths or live births were similar with respect to urban/rural residency and prenatal smoking status; however, those with fetal deaths were more likely to be unmarried, be > 35 years of age, have less than a high school education, have used alcohol prenatally, or be Medicaid recipients (Table 1). Women with fetal deaths were also slightly more likely to be of nonwhite race/ethnicity, and less likely to have had a prior pregnancy or birth. Similar proportions of women whose pregnancies ended in fetal deaths (18%) and live births (17%) lived within 0.5 miles of a hazardous waste site [OR = 1.06; 95% confidence interval (CI), 0.90–1.25] (Table 2). Fetal death was not associated with close maternal residential proximity to a hazardous waste site, nor were significantly increased ORs observed for early (OR = 0.92; 95% CI, 0.39–2.17) or late (OR = 0.99; 95% CI, 0.80–1.23) fetal death (Table 2). These results were similar when analyses were restricted to women with proven fertility (no prior fetal losses and at least one prior live birth), urban or rural area of residence, and to the limited extent possible with these data, when stratified by the reported presence of any congenital anomaly on the fetal death or birth certificate records (data not shown).

Among women who had lived > 5 years at their residence at the time of delivery or fetal death, the OR for fetal death associated with living within 0.5 miles, relative to those living > 5 miles, of a hazardous waste site was 0.80 (95% CI, 0.57–1.11; data not shown). Results were similar for all proximities among women in this group. Among women who had lived < 12 months at their residence at the time of delivery or fetal death, the ORs ranged from 1.39 (95% CI, 0.96–2.00) for residence within > 0.5 mile to 1 mile of the nearest site, to 1.26 (95% CI, 0.86–1.83) for residence within > 2 to 5 miles.

Living within 2 miles of a high-priority site was associated with slightly but not statistically significantly increased risks of fetal death (OR = 1.11; 95% CI, 0.97–1.27 for those living > 1 mile to 2 miles; OR = 1.09; 95% CI, 0.95–1.25 for those living within 1 mile), and we observed no suggestion of a trend with decreasing distance (Table 3). None of the close-proximity categories to a low-priority site were associated with increased risk of fetal death. We also observed no associations with close residential proximities to hazardous waste sites with contaminated water, soil, or air, or with proximity to sites containing solvents or metal. However, fetal death risk was modestly increased among women residing within 5 miles of a pesticide-contaminated site, with ORs starting at 1.13 (95% CI, 1.05–1.22) for women within 2–5 miles, and increasing to 1.28 (95% CI, 1.13–1.46) for those residing within 1 mile. Reanalysis of this relationship using distance as a continuous variable indicated a 1% decrease in the OR with each additional mile of distance (*p* < 0.05). We observed no significant risk of fetal death among women living near a site containing a radioactive substance except for those residing > 2 to 5 miles away (OR = 1.14; 95% CI, 1.02–1.27); however, the OR for women within 2 miles was 0.87 (95% CI, 0.71–1.09).

## Discussion

The causes of fetal death are not clearly understood and in most cases are unknown (Cnattingius and Stephansson 2002). In developed countries, 10–25% of fetal deaths are estimated to be caused by infection (Gibbs 2002; Goldenberg and Thompson 2003), and approximately 25% have been attributed to genetic or metabolic causes (Wapner and Lewis 2002). Other causes include isoimmunization, placental abruption, maternal chronic diseases such as diabetes and hypertension, umbilical cord accidents (Cnattingius and Stephansson 2002; Simpson 2002), and maternal risk factors such as smoking (Wu et al. 1998), advanced age, and obesity (Cnattingius and Stephansson 2002).

Researchers evaluating possible associations between fetal deaths and environmental factors have used various methods and definitions with varied results. Because of the complex interactive effects of various exposures and exposure routes and the timing and duration of these exposures, currently available methods of studying the associations between environmental exposures and pregnancy outcomes are fairly insensitive, making measurement of these associations difficult (Hertz-Picciotto et al. 1996). Events such as the reported high rate of pregnancy loss among women living near the Love Canal (New York) chemical waste site in the late 1970s (New York State Department of Health 1978) have raised public concern about possible reproductive effects of hazardous waste sites, and the use of existing population-based public health data in epidemiologic studies (such as we have done here) is appropriate and necessary as more accurate methods of assessment are developed.

Overall, our results provide insufficient evidence to confirm or exclude the possibility of a relationship between fetal death occurrence and residential proximity to toxic waste sites. Lack of a significant association has been observed in some (Baker et al. 1988; Elliott et al. 2001; Fielder et al. 2000; Sosniak et al. 1994) but not all (Bhopal et al. 1999; Brender et al. 2003; Dummer et al. 2003; Kharrazi et al. 1997) previous studies investigating this relationship, albeit with various methods and exposures (including evaluations of specific landfills or industrial sites). Similarly mixed results have been reported by researchers evaluating the risk of fetal malformation (one important cause of fetal death) among women residing near hazardous waste sites (Croen et al. 1997; Dodds and Seviour 2001; Dolk et al. 1998; Elliott et al. 2001).

We did observe, however, that close maternal residence to a pesticide-contaminated hazardous waste site may increase the risk of fetal death. Residential exposure to pesticides in hazardous waste sites may occur via water contamination, inhalation or ingestion of contaminated dust, ingestion of locally grown contaminated produce, or dermal contact and absorption of chemicals from contaminated particles. Pesticides may affect the developing fetus or a mother’s ability to carry the fetus to an age of viability. Exposure to pesticides may result in somatic cell mutations in the fetus (Hodgson and Levi 1996) or cause hormonal or immune function changes in the fetus (Colborn et al. 1993) or mother (Ahmed 2000; Casale et al. 1998; Stiller-Winkler et al. 1999). Results of previous studies have suggested that maternal exposure to pesticides may be associated with an increased risk of fetal malformations (Shaw et al. 1999; Weidner et al. 1998) and of associated fetal death (Bell et al. 2001), and self-reported home pesticide exposure has previously been associated with stillbirth (Pastore et al. 1997), as has maternal employment in an agricultural occupation (Arbuckle and Sever 1998; McDonald et al. 1988; Vaughan et al. 1984). Early animal studies have also indicated that parental exposure to chemicals and radiation induces anomalies in mice (Nomura 1982), suggesting that fetal loss due to congenital anomalies may be plausibly caused by residing near hazardous waste sites containing chemicals or radioactive material. Our ability to examine fetal deaths due to the presence of malformations was quite limited, but we observed that the risks of fetal death in relation to close proximity to hazardous waste sites did not differ by the presence or absence of malformations as noted in the vital records.

Although we observed a modestly increased fetal death risk among women living > 2 to 5 miles from a site containing radioactive substances, the lack of an association for those residing in the closest proximity category (within 2 miles) and the small numbers of subjects available for these subanalyses suggest that this may be attributed to chance or to the effects of unexamined factors such as topography, water drainage, or wind patterns. Our inability to account for these factors was a limitation in all of our analyses.

As summarized by Vrijheid (2000) in a review of epidemiologic studies of possible associations of health effects and residential proximity to hazardous waste sites, most prior studies evaluated specific contaminated sites and their surrounding communities rather than all sites and populations within a large area, as we have done. Although our study does not share some limitations of many single-site studies such as small sample size, the recall or reporting bias that may be present in studies prompted by public concern over perceived hazards, or dependence on self-reported exposure measurements, it has many limitations. We have no direct measurement of hazardous exposures, and assumed a general regional effect (i.e., that the level of toxicant exposure would be directly related to proximity to a hazardous waste site). This is a limitation of most previous studies of possible associations between adverse pregnancy outcomes and environmental exposures (Baibergenova et al. 2003; Vrijheid 2000). A few recent studies have directly measured pesticide levels in biological or environmental specimens in relation to distance from putative exposure sources. Close residential proximity to a pesticide source was associated with increased pesticide levels in blood (Gaffney et al. 2005), carpet dust (Ward et al. 2006), and indoor and outdoor air (Kawahara et al. 2005). Blood lead levels (Willmore et al. 2006) and some levels of polycyclic aromatic hydrocarbon urinary metabolites were greater among residents living in closer proximity to putative point sources (Bouchard et al. 2001), and volatile organic compounds measured in indoor and outdoor air samples of homes close to a factory were greater than those from homes farther away (Jo and Oh 2001). In addition to these positive studies, we also identified one study reporting that polychlorinated biphenyl (PCB) levels in cord bloods of infants born to mothers living near a PCB-contaminated Superfund site were not associated with distance to the site (Choi et al. 2006). Although it was beyond the limited scope of our own exploratory study to directly measure exposures, the direction of most previous reports suggests that, particularly for pesticides, close proximity to a known contamination source may be associated with increased toxicant exposure.

Hazardous waste sites also typically contain many different chemicals, with different routes of exposures or contaminated media, so the effect of a specific exposure on the health outcome being investigated is difficult to determine. Although we examined associations by type of chemical at a site and by type of contaminated media to address some of these problems, we also lacked information on the duration, route, and dose of exposures, or reliable data regarding parental occupational exposures.

Another limitation to our study was that maternal residence in the vital records may not indicate where a woman actually lived during the relevant part of her pregnancy. Estimates of residential mobility among pregnant women range from about 12% (Fell et al. 2004) to 20% (Khoury et al. 1988), with some variation by age, race, socioeconomic status, smoking status, and other factors. To the extent that residential proximity to a hazardous waste site was misclassified nondifferentially among cases and controls, our results would have been biased toward the null. To the extent that some of these characteristics were also related to the outcome of fetal death, such misclassification may also have biased our results in other ways. Presumably, an analysis limited to women who resided at the address noted on the vital record for at least 12 months before delivery or fetal death would provide a more accurate assessment of the fetal death–proximity relationship, and would partly address the issue of timing of exposure (Hertz-Picciotto et al. 1996). However, we observed only a slightly increased OR that was not statistically significant when we restricted our analysis to these women.

Our study was also limited by the accuracy of the vital record information, especially because fetal deaths, particularly at an early gestational stage, have been underreported (Goldhaber 1989; Harter et al. 1986). In a previous Washington State study, researchers compared fetal death records with records from 16 hospitals and observed that although 92% of all fetal deaths of at least 20 weeks gestation were reported in the vital records, the level of completeness varied by gestational length, from 78% for those of 20–23 weeks gestation, to 96% for those of 36–39 weeks gestation (Harter et al. 1986). This underreporting of early fetal death is another factor that may have biased our results, especially if that under-reporting was also associated with other factors related to fetal death. Our study may have been affected by other inaccuracies in vital records data. Although the results of a recent validation study comparing information on fetal death records with medical record data for women at a tertiary care hospital in Washington State showed good agreement on many of the variables assessed, including history of prior pregnancies and chronic maternal conditions such as hypertension and diabetes (Lydon-Rochelle et al. 2005a), there was poorer agreement on several variables (e.g., placental cord conditions), and up to 25% of fetal death records were missing data for some of the factors examined. A similar evaluation of selected medical complications on birth records indicated underreporting of several conditions such as gestational diabetes (Lydon-Rochelle et al. 2005b), so we had limited ability to control for any relevant confounding. Also, although vital records contained information on the number of prior pregnancies and births, we were unable to reliably distinguish timing and types of prior pregnancy loss. An additional consideration in our study is that a residential geocode was less likely to have been determined for women with fetal deaths (75%) than those with live births (86%), and it is likely that at least some of the characteristics associated with fetal death (such as smoking and unmarried status) are also associated with use of a post office box or having address information that could not be geocoded for other reasons. To the extent these characteristics may also be associated with proximity to hazardous waste sites (e.g., if poor or unmarried women were more likely to live closer to them) this disproportionate exclusion of women with fetal deaths is another factor that may have biased our results toward the null.

Inaccuracies in the coordinates obtained from addresses in the vital records may have resulted in misclassification; however, some research indicates that geocoding of addresses using commercial software such as we employed was generally quite accurate when compared to the “gold standard” of positions obtained by using global positioning system receivers (Bonner et al. 2003). The median difference in positions obtained by the two methods was small, at 38 m (∼ 0.02 mile), suggesting that the accuracy of geocoding in our study in terms of locating a specific address was probably quite good. Misclassification may also have occurred, however, because of errors in the geocoordinates for toxic waste sites, which were obtained using several methods and which we were unable to validate.

We also had access to information only about fetal deaths that occurred after at least 20 weeks gestation, and we were not able to explore whether close residential proximity to a hazardous waste site was associated with earlier pregnancy loss that may have been caused by the occurrence of severe anomalies or other conditions incompatible with maintaining pregnancy beyond very early stages. However, there did not appear to be any differences in the associations observed for early (20–27 weeks) versus later fetal death (≥ 28 weeks gestation).

Our finding of no overall significant association between fetal death and maternal residential proximity to hazardous waste sites should not delay clean-up efforts or research to develop more accurate exposure measurements. Our finding of an increased risk associated with close residential proximity to pesticide-containing sites suggests an important focus for future work, particularly in view of other evidence supporting associations of pesticide exposure and adverse reproductive outcomes (Arbuckle and Sever 1998; Perera et al. 2005).

## Figures and Tables

**Table 1:** Maternal and infant characteristics of fetal death cases and controls with live births, Washington State 1987–2001 [no. (%)].

Characteristic	Cases^a^ (*n* = 5,302)	Controls^a^ (*n* = 61,455)
Maternal		
Age (years)		
< 20	652 (13)	6,524 (11)
20–24	1,141 (22)	15,327 (25)
25–29	1,332 (26)	17,491 (28)
30–34	1,112 (22)	14,384 (23)
35–39	704 (14)	6,434 (10)
40–44	211 (4)	1,195 (2)
≥ 45	11 (< 1)	60 (< 1)
Education^b^		
< High school	762 (23)	7,187 (18)
High school	1,088 (32)	13,054 (32)
≥ College	1,501 (45)	20,672 (51)
Marital status		
Married	3,391 (66)	45,295 (74)
Unmarried	1,723 (34)	16,032 (26)
Medical insurance^b^		
Medicaid/sponsored	1,430 (31)	15,537 (26)
HMO/commercial	2,429 (53)	34,996 (59)
Other	741 (16)	8,651 (15)
Urban/rural residence^c^		
Urban	4,643 (87)	53,536 (87)
Rural	659 (12)	7,919 (13)
Smoked prenatally		
No	3,561 (83)	49,467 (84)
Yes	735 (17)	9,664 (16)
Prenatal alcohol use^d^		
No	3,741 (91)	51,039 (98)
Yes	351 (9)	1,250 (2)
No. of prior pregnancies		
0	1,857 (37)	19,683 (32)
1	1,109 (22)	17,124 (28)
2	791 (16)	11,342 (18)
≥ 3	1,199 (24)	13,306 (22)
No. of prior live births		
0	2,473 (56)	25,373 (42)
1	1,117 (25)	19,840 (33)
2	500 (11)	9,619 (16)
≥ 3	342 (8)	6,220 (10)
Race/ethnicity		
White	3,541 (72)	46,386 (78)
African American	398 (8)	2,416 (4)
Native American	131 (3)	1,211 (2)
Asian/Pacific Islander	335 (7)	4,041 (7)
Hispanic	544 (11)	5,609 (9)
Other	1 (< 1)	30 (< 1)
Infant		
Sex		
Male	2,771 (53)	31,444 (51)
Female	2,498 (47)	30,011 (49)

HMO, health maintenance organization.

a Numbers may not add to totals because of missing data.

b Data available for 1992–2001 only.

c Based on census tract of residence.

d Data available for 1989–2001 only.

**Table 2:** Fetal death in relation to maternal residential proximity to nearest hazardous waste site during pregnancy, by gestational age, Washington State, 1987–2001.

				Early fetal death (20–28 weeks) (*n* = 1,827)		Late fetal death (> 28 weeks) (*n* = 2,076)	
Distance to nearest site (miles)	Controls (*n* = 59,097) %	Cases (*n* = 3,903) %	Any fetal death (≥ 20 weeks) OR^a^ (95% CI)	%	OR^a^ (95% CI)	%	OR^a^ (95% CI)
> 5	5	5	1.0 (reference)	4	1.00 (reference)	5	1.00 (reference)
> 2– ≤ 5	21	20	0.98 (0.83–1.15)	19	1.06 (0.45–2.52)	21	0.95 (0.77–1.17)
> 1– ≤ 2	32	31	1.00 (0.85–1.17)	32	1.11 (0.48–2.54)	30	0.90 (0.73–1.11)
> 0.5– ≤ 1	25	25	1.02 (0.87–1.19)	26	1.14 (0.61–3.42)	24	0.91 (0.74–1.12)
≤ 0.5	17	18	1.06 (0.90–1.25)	18	0.92 (0.39–2.17)	19	0.99 (0.80–1.23)

a Adjusted for maternal age, prenatal smoking status, and number of prior pregnancies.

**Table 3:** Risk^a^ of fetal death in relation to maternal residential proximity to nearest hazardous waste site, by selected site characteristics, Washington State 1987–2001 [OR (95% CI)].

	Distance from maternal residence to nearest site (miles)			
Site characteristics	> 5	> 2– ≤ 5	> 1– ≤ 2	≤ 1
Site priority				
High-priority site^b^	1.0 (reference)	1.01 (0.89–1.14)	1.11 (0.97–1.27)	1.09 (0.95–1.25)
Low-priority site^b^	1.0 (reference)	0.95 (0.84–1.08)	0.97 (0.86–1.10)	1.00 (0.88–1.13)
Type of contaminated media				
Water (any)	1.0 (reference)	0.96 (0.82–1.11)	1.02 (0.88–1.18)	1.01 (0.88–1.17)
Drinking water	1.0 (reference)	0.97 (0.89–1.04)	1.05 (0.96–1.15)	1.01 (0.90–1.13)
Soil and sediment	1.0 (reference)	0.99 (0.85–1.16)	1.02 (0.88–1.19)	1.04 (0.90–1.21)
Air	1.0 (reference)	0.92 (0.85–1.01)	0.97 (0.89–1.07)	0.92 (0.83–1.01)
Type of contaminant				
Solvents	1.0 (reference)	0.91 (0.83–1.01)	1.02 (0.92–1.12)	0.93 (0.83–1.05)
Metals	1.0 (reference)	0.91 (0.81–1.03)	0.94 (0.83–1.05)	0.95 (0.85–1.07)
Pesticides	1.0 (reference)	1.13 (1.05–1.22)	1.17 (1.06–1.29)	1.28 (1.13–1.46)
	> 10	> 5 – ≤ 10	> 2 – ≤ 5	≤ 2
Type of contaminant				
Radioactive substances	1.0 (reference)	1.04 (0.96–1.13)	1.14 (1.02–1.27)	0.87 (0.71–1.07)

a Adjusted for maternal age, prenatal smoking status, and number of prior pregnancies.

b Data for high-priority sites also adjusted for distance to nearest low-priority site and vice versa.
